# Supplementary material for: European and Mediterranean Myzocallidini Aphid Species: DNA Barcoding and Remarks on Ecology with Taxonomic Modifications in An Integrated Framework
Source: Insects. 2022 Nov 1;13(11):1006. doi: 10.3390/insects13111006 (PMC9693350; doi:10.3390/insects13111006)
Supplement: Supplementary file 1 [file insects-13-01006-s001.zip › Table S1 - Host plants, sampling date, geographical coordinates, voucher numbers, Bold seq ID and Gb accession number of Myzocallis spp. analysed.pdf]

Table S1. Host plants, sampling date, geographical coordinates, voucher numbers, BIN and Genebank accession number of *Myzocallis* spp. analysed.

Species	Host plant	Sampling date	Location	long/lat	Voucher number	GeneBank accession
Myzocallis carpini	*Carpinus betulus*	31 May 2017	Piemonte, Italy	49.35/1.02	MYZ/728	OP143871
M. carpini	*C. betulus*	23 Oct 2017	Piemonte, Italy	45.05/7.68	MYZ /753	OP143872
M. occidentalis	*Quercus. pyrenaica*	5 May 2018	Castile and León, Spain	42.66/-5.47	MYZ /811	OP143877
M. occidentalis	*Q. pyrenaica*	11 Nov 2017	Castile and León, Spain	42.67/-5.47	MYZ /767	OP143880
M. occidentalis	*Q. pyrenaica*	11 Nov 2017	Castile and León, Spain	42.66/-5.48	MYZ /768	OP143879
M. occidentalis	*Q. pyrenaica*	21 Nov 2017	Castile and León, Spain	42.66/-5.46	MYZ /776	OP143878
M. glandulosa	*Q. ithaburensis*	15 May 2019	Neve Yaar, Israel	32.70/35.17	MYZ /832	OP143890
M. schreiberi	*Quercus. x crenata*	12 Oct 2017	Piemonte, Italy	45.08/7.63	MYZ /748	OP143885
M. schreiberi	*Q. suber*	27 Nov 2017	Sicily, Italy	38.11/13.37	MYZ /765	OP143887
M. schreiberi	*Q. ilex*	1 Apr 2019	Sicily, Italy	37.21/14.84	MYZ /823	OP143886
M. schreiberi	*Q. suber*	11 Apr 2019	Sicily, Italy	37.10/14.50	MYZ /826	OP143884
M. schreiberi	*Q. suber*	11 Apr 2019	Sicily, Italy	37.09/14.50	MYZ /828	OP143881
M. macrolepidis	*Quercus macrolepis*	28 May 2017	Apulia, Italy	39.92/18.37	MYZ /713	OP143892
M. macrolepidis	*Q. macrolepis*	11 May 2018	Apulia, Italy	39.92/18.38	MYZ /790	OP143893
M. boerneri	*Quercus cerris*	14 June 2017	Sicily, Italy	37.77/15.09	MYZ /719	OP143873
M. boerneri	*Q. cerris*	14 June 2017	Sicily, Italy	37.73/15.10	MYZ /725	OP143876
M. coryli	*Corylus avellana*	26 May 2017	Sicily, Italy	37.79/14.82	MYZ /712	OP143869
M. coryli	*C. avellana*	14 Jun 2017	Sicily, Italy	37.83/14.77	MYZ /724	OP143870
M. coryli	*C. avellana*	26 Dec 2017	Franklin, South Africa	-30.35/29.46	MYZ /779	OP143866
M. coryli	*C. avellana*	19 Jun 2017	Copenaghen, Denmark	55.68/12.56	MYZ /815	OP143867
M. coryli	*C. americana*	28 Jun 2018	Sicily, Italy	38.11/13.77	MYZ /817	OP143868
M. castanicola	*Q. cerris*	17 May 2007	Sardinia, Italy	40.33/8.95	MYZ/699	OP143817
M. castanicola	*Q. petraea*	26 May 2018	Piemonte, Italy	45.08/7.33	MYZ/803	OP143815
M. castanicola	*Q. petraea*	31 Oct 2017	Piemonte, Italy	45.09/7.34	MYZ/749	OP143813
M. castanicola	*Quercus pubescens*	2 Jun 2018	Piemonte, Italy	45.10/7.45	MYZ/805	OP143814
M. castanicola	*Q. pyrenaica*	21 Nov 2017	Castile and León, Spain	42.66/-5.47	MYZ/774	OP143919

M. castanicola	*Q. pyrenaica*	15 May 2018	Castile and León, Spain	42.66/-5.47	MYZ/799	OP143918
M. castanicola	*Q. pyrenaica*	5 May 2018	Castile and León, Spain	42.5/-5.49	MYZ/812	OP143816
M. leclanti	*Castanea sativa*	13 Jun 2017	Sicily, Italy	37.69/14.93	MYZ/717	OP143921
M. leclanti	*C. sativa*	14 Jun 2017	Sicily, Italy	37.69/14.93	MYZ/720	OP143920
M. leclanti	*C. sativa*	17 May 2017	Piemonte, Italy	45.07/7.36	MYZ/746	OP143922
M. miricae	*Myrica gale*	20 Sept 2019	Galicia, Spain	43.00/-8.92	MYZ/822	OP143811
M. cocciferina	*Quercus coccifera*	15 Jun 2017	Sicily, Italy	36.93/14.53	MYZ/727	OP143809
M. cocciferina	*Q. coccifera*	11 May 2018	Apulia, Italy	40.94/16.94	MYZ/795	OP143908
M. cocciferina	*Q. ilex x coccifera*	25 May 2017	Sicily, Italy	37.51/15.08	MYZ/709	OP143905
M. cocciferina	*Q. ilex*	5 Jul 2017	Siclily, Italy	37.88/15.13	MYZ/741	OP143904
M. cocciferina	*Q. ilex x coccifera*	5 May 2018	Sicily, Italy	37.51/15.08	MYZ/782	OP143906
M. mediterranea	*Quercus pubescens*	17 Jun 2017	Sicily, Italy	37.83/15.13	MYZ/740	OP143896
M. mediterranea	*Q. pubescens*	8 May 2018	Sicily, Italy	37.57/15.11	MYZ/783	OP143895
M. mediterranea	*Q. pubescens*	8 May 2018	Sicily, Italy	37.62/15.07	MYZ/784	OP143894
M. mediterranea	*Q. pubescens*	8 May 2018	Sicily, Italy	37.58/15.11	MYZ/785	OP143897
M. komareki	*Q. macrolepis*	28 May 2017	Apulia, Italy	39.92/18.38	MYZ/714	OP143903
M. komareki	*Q. cerris*	17 May 2017	Piemonte, Italy	45.05/7.37	MYZ/735	OP143901
M. komareki	*Q. petraea*	31 Oct 2017	Piemonte, Italy	45.09/7.35	MYZ/754	OP143902
M. komareki	*Q. trojana*	27 Nov 2019	Sicily, Italy	38.11/13.37	MYZ/793	OP143898
M. walshii	*Quercus rubra*	31 May 2017	Sicily, Italy	45.08/7.63	MYZ/733	OP143928
M. walshii	*Q. rubra*	22 Jul 2017	Katowice, Poland	50.26/19.03	MYZ/760	OP143927
Tuberculatus eggleri	*Q. pubescens*	13 Jun 2017	Sicily, Italy	37.66/14.96	TUB/716	OP143923
T. neglectus	*Q. petraea*	31 Oct 2017	Piemonte, Palermo	45.09/7.35	TUB/751	OP143924
T. remaudieri	*Q. pyrenaica*	21 Nov 2017	Castle and León, Spain	42.66/-5.47	TUB/771	OP143925
Apulicallis trojanae	*Q. trojana*	10 May 2018	Apulia, Italy	40.67/17.22	APU/788	OP143888
Siculaphis vittoriensis	*Q. ilex*	1 Apr 2019	Sicily, Italy	37.21/14.84	SIC/823	OP143889
Hoplocallis ruperti	*Q. macrolepis*	27 Nov 2019	Sicily, Italy	38.11/13.37	HOP/762	OP143926
